# Early feeding with hyperglucidic diet during fry stage exerts long-term positive effects on nutrient metabolism and growth performance in adult tilapia (*Oreochromis niloticus*)

**DOI:** 10.1017/jns.2020.34

**Published:** 2020-09-07

**Authors:** Suksan Kumkhong, Lucie Marandel, Elisabeth Plagnes-Juan, Vincent Veron, Stephane Panserat, Surintorn Boonanuntanasarn

**Affiliations:** 1 School of Animal Technology and Innovation, Institute of Agricultural Technology, Suranaree University of Technology, Muang, Nakhon Ratchasima 30000, Thailand; 2 INRAE, Université de Pau et des Pays de l'Adour, E2S UPPA, NuMéA, F-64310 Saint-Pée-sur-Nivelle, France

**Keywords:** Nile tilapia, Nutrient programming, Growth performance, Early feeding, Gene expression, Glucose metabolism

## Abstract

The present study aimed to investigate nutritional programming of carbohydrate metabolism in Nile tilapia. Early nutritional intervention stimulus was achieved by feeding fry with high-protein/low-carbohydrate (HP/LC) or low-protein/high-carbohydrate (LP/HC) diet since first feeding for 4 weeks, and the effect of nutritional stimulus on carbohydrate and its related metabolism was evaluated through the adult stage. Our findings indicated that at week 1, LP/HC diet-fed fry had lower levels of mRNA for genes coding gluconeogenesis and amino acid catabolism and higher levels of *hk*2 (*P* < 0⋅05). As expected, in adult tilapia, although LP/HC diet-fed fish had poorer growth (end of stimulus), the fish showed compensatory growth. There were permanent effects of early high-carbohydrate (HC) intake on several parameters, including (1) modulating hepatic composition, (2) increased muscle glycogen, (3) lower levels of enzymes involved in amino acid catabolism and (4) higher levels of glycolytic enzymes in glycolysis. Finally, HP/LC diet- and LP/HC diet-fed fish were challenged with different dietary carbohydrate levels. Irrespective of challenging diets, the early HC stimulus had significant effects on adult tilapia by (1) promoting utilisation of glucose, which had protein-sparing effects for better growth, (2) inducting lipogenesis and (3) decreasing amino acid catabolism. Taken together, for the first time, we demonstrated that early HC feeding was effective for positive nutritional programming of metabolism in Nile tilapia (an omnivorous fish). It led to the improvement of growth performance in adult fish associated with early feeding, which is linked to a better ability to use glucose, to induce lipogenesis, and to suppress amino acid catabolism.

## Introduction

Fish nutrition is vital for the development of a sustainable aquaculture^(1)^. Recent studies in fish nutrition have focused on exploring the potential use of new feed ingredients and on developing a novel concept (nutritional programming) to modify specific metabolic functions for better use of new aquafeeds^(2)^. Nutritional programming is based on the fact that early feeding may have a long-term impact on metabolic processes in later life^(3,4)^. In fish, nutritional programming has been tested to investigate the possibility of early nutritional history to be applied for tailoring metabolic pathways for better use of new ingredient-based diets^(2,5–9)^.

Although carbohydrates are not an essential nutrient in fish, they are incorporated into diets to produce low-cost diet as well as diets with a lower level of dietary proteins. The metabolic use of dietary carbohydrates has been intensively investigated in reared fish. For example, nutritional factors affecting carbohydrate metabolism were demonstrated in a number of fish species with distinct feeding habits from the carnivorous fish with low capacity to use dietary carbohydrates up to herbivorous and omnivorous fish with a high capacity to use dietary carbohydrates^(10–18)^. To improve the metabolic use of dietary carbohydrates, nutritional programming for carbohydrate metabolism has been recently conducted in different fish species. Nutritional programming has been successfully demonstrated using the first-feeding stage in carnivorous fish, including rainbow trout (*Oncorhynchus mykiss*)^(19,20)^, Siberian sturgeon (*Acipenser baerii*)^(21,22)^, sea bass (*Dicentrarchus labrax)*^(23)^ and gilthead seabream (*Sparus aurata*)^(24,25)^. However, nutritional programming in omnivorous fish has been reported in few studies on zebrafish (*Danio rerio*)^(26–28)^ and only one study on Nile tilapia (*Oreochromis niloticus*)^(29)^.

Nile tilapia (*Oreochromis niloticus*) is an omnivorous fish that can efficiently use high levels of dietary carbohydrates^(16,30,31)^. Numerous studies on nutrition have been conducted on Nile tilapia because it is an economically important freshwater fish. Moreover, Nile tilapia is the second most important cultured fish after the common carp worldwide^(1)^. As a model of omnivorous fish, nutritional regulation of glucose metabolism was demonstrated in previous studies, which revealed that intake of high dietary carbohydrates (up to 40 %) was associated with better growth performance and down-regulation of gluconeogenesis and an increase in glycogen and lipid deposits in the liver and muscle^(18)^, compared with carnivorous fish^(16)^.

Because early feeding with a high level of carbohydrates can have positive long-term effects on growth performance and glucose metabolism^(19,20,24,26–28,32,33)^, it is important to test this hypothesis of direct and long-term impacts of early feeding with high levels of carbohydrates in an omnivorous fish species (i.e. the Nile tilapia). Thus, for the first time, we aimed to evaluate the direct effects of high-carbohydrate (HC) diet by measuring the mRNA levels of genes involved in intermediary metabolism in tilapia fry. The long-term effects of nutritional history (early HC intake at first feeding) were determined up to the adulthood stage by analysing intermediary metabolism at molecular and enzymatic levels in the liver and muscle, determining plasma metabolites and evaluating tissue compositions before and after a final dietary challenge with an HC diet.

## Materials and methods

### Experimental fish and diets, experimental design and fish culture

All experimental protocols regarding the fish were approved by the Ethics Committee of Suranaree University of Technology, Animal Care and Use Committee (approval no. A-18/2562). Nile tilapia fry (*O. niloticus*) used in the present study was obtained from a broodstock that was cultured at the University Farm, Suranaree University of Technology, Nakhon Ratchasima, Thailand. Nile tilapia broodstock (0⋅8–1⋅2 kg) was cultured in an earthen pond (800 m^2^) and fed with a commercial feed (30 % crude protein (CP) + 4 % crude fat (CF) at 3 % body weight) at 9:00 and 16:30 daily. Table 1 details the ingredients of the two diets for the early feeding stage, i.e. high-protein/low-carbohydrate (HP/LC) and low-protein/high-carbohydrate (LP/HC) diets, and of the two diets for the final challenge, i.e. the medium-carbohydrate diet (CHO-M; 37 % of carbohydrates) and HC diet (CHO-H; 67 % of carbohydrates). The proximate composition including moisture, CP, CF, crude fibre, ash and gross energy was analysed according to the standard method by the Association of Official Analytical Chemists (AOAC)^(34)^ (Table 1). To prevent the confounding effect associated with sex dimorphism, all male fish were used in the present study. Therefore, HP/LC and LP/HC diets were supplemented with 17 α-methyltestosterone (17MT) at 60 mg/kg^(18)^. Currently, all male tilapia, which were produced using feeding fish fry 17MT, have been commercially cultured for global consumption, and several reports have revealed no accumulation of 17MT in fish flesh at harvesting size^(35)^.

**Table 1.** Ingredients and chemical composition (g/kg) of the two stimulus diets (diets at first feeding) and the two challenge diets (diets for the final challenge)

Ingredients	Stimulus diets		Challenge diets	
	HP/LC	LP/HC	CHO-M	CHO-H
Fish meal	860	360	350	140
Soybean meal	–	–	300	60
Rice flour	100	560	150	700
Rice bran	–	–	180	30
Soybean oil	20	60	–	40
Fish premix^a^	20	20	–	10
Dicalcium phosphate	–	–	20	20
Proximate composition (g/kg dry weight)				
Dry matter	957⋅8	956⋅6	957⋅7	957⋅3
Protein	492⋅6	247⋅1	356⋅7	154⋅9
Fat	96⋅9	99⋅2	69⋅0	64⋅8
Fibre	5⋅8	4⋅8	28⋅9	8⋅6
Ash	238⋅1	108⋅1	129⋅2	60⋅3
NFE^b^	166⋅7	540⋅9	374⋅0	668⋅8
Gross energy (kJ/g)	17⋅8	18⋅6	17⋅6	17⋅2

a Vitamin and trace mineral mix provided the following (IU/kg or g/kg diet): biotin, 0⋅25 g; folic acid, 0⋅003 g; inositol, 0⋅25 mg; niacin, 0⋅0215 g; pantothenic acid, 0⋅03 g; vitamin A, 5000 IU; vitamin B1, 0⋅0025 g; vitamin B2, 0⋅0012 g; vitamin B6, 0⋅0075 g; vitamin B12, 0⋅00005 mg; vitamin C, 1 g; vitamin D3, 1000 IU; vitamin E, 100 IU; vitamin K, 0⋅008 g; copper, 0⋅02 g; iron, 0⋅2 g; selenium, 0⋅3 mg; zinc, 0⋅32 g.

b Nitrogen-free extract = Dry matter − (CP + crude lipid + crude fibre + ash).

**Fig. 1.**
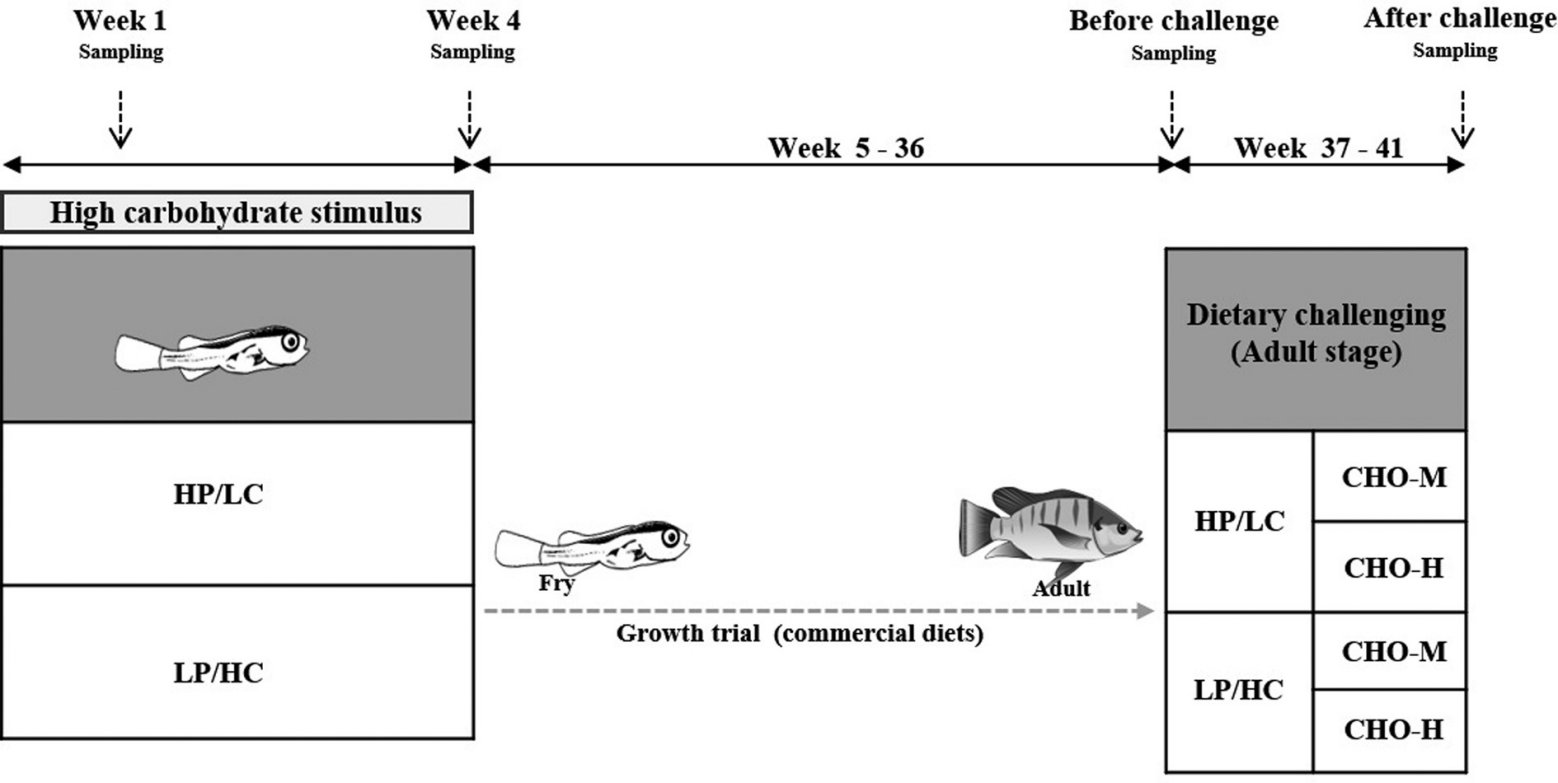
Experimental plan for the HC stimulus (history) and the dietary carbohydrate challenge test. Nile tilapia fry were fed either a high-protein/low-carbohydrate (HP/LC) or a low-protein/high-carbohydrate (LP/HC) diet at the first-feeding stage for 4 weeks. At week 1, fry was sampled to examine the expression of genes involved in carbohydrate and its related metabolism. See Table 1 for the list of genes and their respective primers. At the end of stimulus (4 weeks), fry were sampled to determine the glycogen and triacylglycerol contents and the expression of genes that are involved in carbohydrate and its related metabolism. Subsequently, the fish were cultured for a growth trial during weeks 5–36 after stimulus. During the growth trial, commercial diets (weeks 5–8, 40 % CP + 8 % CF; weeks 9–20, 32 % CP + 4 % CF; weeks 21–36, 30 % CP + 4 % CF) were used for feeding. Before a challenge test (week 36), the fish were sampled to examine the effects of stimulus history. During weeks 37–41, the fish were subjected to a challenge test with different dietary carbohydrate levels (37 % carbohydrates, CHO-M; 67 % carbohydrates, CHO-H). Fish sampling was performed before (week 36) and after (week 41) the challenge to determine blood metabolites, muscle and hepatic chemical composition, and expression of metabolic genes.

An illustrative view of the experimental design is shown in Fig. 1. To investigate the effects of the early HC diet (namely the ‘stimulus’ phase), a completely randomised design with the two first-feeding diets (HP/LC and LP/HC diets) was employed using six replicates (cages). In total, 1200 fry (9–10 mg) were randomly distributed into twelve cages (40 × 40 × 60 cm^3^). To exclude the possible effects of the environment during the stimulus phase, twelve cages were located in one cement pond (2 × 2 × 0⋅8 m^3^) (six replicates; 100 fry/replicate), and an HP/LC or LP/HC diet was fed for 4 weeks daily at 09:00, 11:00, 13:00, 15:00 and 17:00, as described previously^(18)^. Both diets were well accepted by the fish for 4 weeks. Subsequently, both experimental fish were continually cultured in cement ponds and fed with a commercial diet *ad libitum* twice daily (at 09:00 and 16:00) up to week 36, and growth performance was determined. During weeks 37–41, for the dietary ‘challenge’ phase, a 2 × 2 factorial design, with the early dietary stimuli (HP/LC and LP/HC diets, namely HP/LC ‘history’ and LP/HC ‘history’) combined with two dietary carbohydrate levels for the dietary challenge (CHO-M and CHO-H), was employed in a completely randomised design using six replicates (cages). Twelve fish from each cement pond replicate were randomly distributed into two cages (80 × 90 × 110 cm^3^) (*n* 6/cage) and fed with either CHO-M or CHO-H diet. Further, growth performance was determined. Throughout the experimental period, dead fish were recorded daily. All thirty experimental fish were cultured under a 12/12 h light/dark cycle in a hatchery. Water and air temperature were determined daily, which were in ranges of 27⋅5–28⋅6°C and 30⋅0–36⋅0°C, respectively. Dissolved oxygen (DO) and pH were recorded weekly using a DO meter and a pH meter, respectively. DO levels of 4⋅2 ± 0⋅4 mg/l (average ± sd) and pH of 7⋅4 ± 0⋅2 (average ± sd), which were acceptable ranges, were observed.

### Fish sampling and blood collections

At week 1 of early dietary stimulus, a pool of three fish per replication (total = 6 replicates) was sampled for analysis of metabolic gene expression. At the end of the early dietary stimulus (4 weeks of feeding), whole bodies of fry (1 fish/replicate; total = 6 replications) were collected to examine levels of glycogen and triacylglycerol (TAG) as well as some metabolic gene expression. For sampling, at 4 h after the last meal (corresponding to the peak of postprandial glycaemia in tilapia; 4–5 h after meal), fish were euthanasized by exposing them to freezing cold water.

At week 36 (before the dietary challenge), six fish per pond were sampled for analysis of blood metabolites and metabolic gene expression as well as chemical composition in the liver and muscle. At 5 h after the last meal (corresponding to the peak of postprandial glycaemia in tilapia), fish were anaesthetized with 10 % clove oil. Subsequently, blood samples were collected from the caudal vein using a hypodermic syringe and mixed with K_2_EDTA (at 1⋅5 mg/ml). Plasma was collected after centrifugation at 3000 × *g* for 10 min at 4°C and stored at −80°C for plasma metabolite determination. Then, liver and muscle tissue samples were obtained and frozen in liquid nitrogen and kept at −80°C for metabolic gene expression and chemical composition analysis according to the method by AOAC^(34)^.

At week 41, to investigate the interactions between early dietary stimuli and the final dietary challenge, fish (two fish/cage) were collected for analysis of metabolic gene expression, and other fish (three fish/cage) were selected for analysis of blood metabolites and chemical composition in the liver and muscle, as described previously for the sampling at week 36.

### Blood metabolite analysis

Plasma metabolites, including glucose, TAG and blood urea nitrogen (BUN), were determined. Plasma glucose was analysed in duplicate according to Trinder's method^(36)^. TAG levels were also determined in duplicate using the glycerol-3-phosphate oxidase-sodium *N*-ethyl-*N*-(3-sulfopropyl)-*m*-anisidine method, as described by Bucolo and David^(37)^. In addition, BUN content was measured in duplicate using a modified indophenol colorimetric method^(38)^.

### Chemical composition and glycogen and TAG analysis

At the end of the early dietary stimulus (week 4), fish were sampled for glycogen and TAG analysis. At weeks 36 (before the challenge) and 41 (after the challenge), the chemical contents in the liver and muscle included moisture, CP, CF and ash, according to AOAC^(34)^. In addition, the hepatic and muscular glycogen contents were determined. The glycogen content was measured using a modified hydrolysis method^(39)^. The sample was ground in 1 M HCl. An aliquot was obtained, neutralised by 5 M KOH, and subsequently centrifuged at 10 000 × *g* at 4°C for 10 min to measure free glucose content. Free glucose was measured using a plasma glucose kit (Glucose RTU; bioMérieux, Marcy-l'Étoile, France), according to the manufacturer's instructions. The other aliquot was boiled at 100°C for 2⋅5 h and then neutralised using 5 M KOH. After 10 min of centrifugation at 10 000 × *g* at 4°C, the total glucose (free glucose + glucose obtained from hydrolysis of glycogen) was analysed using the glucose kit (Glucose RTU). The glycogen content was calculated from the glucose amount after subtracting the total glucose with free glucose levels according to Good *et al*.^(39)^. To analyse TAG, whole bodies of fry were homogenised in liquid nitrogen and 100 mg of the sample was homogenised again in 1 ml of 5 % Igepal in a deionised water solution using a Dounce homogenizer. Samples were heated at 90°C in a water bath for 5 min and subsequently cooled down to room temperature. Then, the heated step was repeated. Subsequently, centrifugation was performed at 10 000 × *g* for 10 min at 4°C to remove any insoluble material, and the supernatants were collected and diluted with deionised water. TAG was measured using a TG plasma kit (Sobioda, Montbonnot, France), following the manufacturer's instructions.

### Total RNA extraction and relative quantification of mRNAs

Real-time reverse transcription–polymerase chain reaction (RT-PCR) was used to measure relative gene expression of intermediary metabolism (glucose, lipid and amino acid metabolism). Samples used for total RNA preparation included the whole bodies of fry (week 1, pool of three fish/replication, *n* 18 per experimental group; week 4, two fish/replication, *n* 12 per experimental group) as well as the liver (50 mg) and muscle (100 mg) (weeks 36 and 41, two fish/replication, *n* 12 per experimental group). Total RNAs were extracted from tissue samples using a TRIzol reagent (Invitrogen, Carlsbad, CA, USA), according to the manufacturer's recommendations. The extracted RNA was quantified by NanoDrop (Thermo Fisher, Madison, WI, USA) and verified on a 1 % agarose gel. To synthesize cDNA, a SuperScript III RNase H Reverse-Transcriptase Kit (Invitrogen) with random primers (Promega, Charbonnières, France) was used with a sample of 1 μg of total RNA (duplicate for each sample, *n* 12 for each treatment group), 100 units of SuperScript III enzyme, and 40 units of an RNase OUT enzyme, following the manufacturer's protocol.

Table 2 presents the primer sequences used in the RT-PCR assays of each metabolic gene^(17,18,40)^. The expression of glucose metabolic genes in the liver was measured, including glycolysis (glucokinase, *gck*; phosphofructokinase, *pfklr*; pyruvate kinase, *pklr*) and gluconeogenesis (glucose-6-phosphatase1 and 2, *g*6*pca*1 and *g*6*pca*2; phosphoenolpyruvate carboxykinase cytosolic, *pck*1; mitochondria, *pck*2). Glucose metabolism in the muscles was analysed by measuring the mRNA levels of glucose transporter (*glut*4) and glycolysis (hexokinase I and II, *hk*1 and *hk*2; phosphofructokinase, *pfkma* and *pfkmb*; pyruvate kinase, *pkma*). Hepatic lipogenic capacities (fatty acid synthase, *fasn*; glucose-6-phosphate dehydrogenase, *g*6*pd*) were examined. In addition, the enzymes involved in amino acid catabolism (glutamate dehydrogenase, *gdh*; alanine aminotransferase, *alat*; aspartate amino transferase, *asat*) were determined. A Roche LightCycler 480 system (Roche Diagnostics, Neuilly-sur-Seine, France) was used for RT-PCR assays of each level of transcript of all metabolic genes. Assays were performed using a reaction mix of 6 μl per sample, and each of them contained 2 μl of a diluted cDNA template (1:40), 0⋅24 μl of each primer (10 μM), 3 μl of LightCycler 480 SYBR® Green I Master Mix (Roche Diagnostics) and 0⋅76 μl of DNase/RNase-free water (5 Prime GmbH, Hamburg, Germany). The PCR protocol was initiated at 95°C for 10 min for the initial denaturation of the cDNA and hot-start Taq polymerase activation, followed by 45 cycles of a three-step amplification program (15 s at 95°C, 10 s at 60–64°C (according to the primer set used) and 15 s at 72°C to extend the DNA). The melting curves were systematically analysed (temperature gradient at 1⋅1°C/s from 65 to 97°C, five acquisitions/1°C) at the end of the last amplification cycle to confirm the specificity of the amplification reaction. Each PCR assay included replicate samples (duplicates of RT and PCR amplification) and negative controls (reverse transcriptase- and cDNA template-free samples). For the analysis of mRNA levels, relative quantification of target gene expression was performed using the Roche Applied Science E-Method^(41)^. The relative gene expression of *ef*1*α* was analysed for the normalisation of the measured mRNA in each tissue because its relative expression was not significantly varied according to the sampling process (data not presented). In all cases, PCR efficiency was calculated from the slope of a standard curve using serial dilutions of cDNA. In all cases, PCR efficiency values were acceptable and ranged between 1⋅8 and 2⋅0.

**Table 2.** List of the primers used for qRT-PCR

Genes	5′/3′ forward primer	5′/3′ reverse primer	Access number
Reference gene			
*ef*1^a^	GCACGCTCTGCTGGCCTTT	GCGCTCAATCTTCCATCCC	AB075952
Liver metabolism			
*gck*	GGGTGGTAGGATTTGGTGTG	TGCTGACACAAGGCATCTTC	XM003451020
*pfklr*	GACGAGCGAGTGGAGAAAAC	TGTCTTGATCCGAGGGAATC	XM003447353
*pklr*	AGGTACAGGTCACCCGTCAG	CATGTCGCCAGACTTGAAGA	XM005472622
*g*6*pca*1	AGCGTTAAGGCAACTGGAGA	AAAAGCTAACAAGGCCAGCA	XM003448671
*g*6*pca*2	CTTCTTCCCCCTTTGGTTTC	AGACTCCTGCAGCTCCCATA	XM013273429
*pck*1	AAGCTTTTGACTGGCAGCAT	TGCTCAGCCAGTGAGAGAGA	XM003448375
*pck*2	TACGTCTTGAGCTCCCGTCT	CCTCCTGGATGATGCAAGTT	XM019354843
*fasn*	AACCTGCTTCTCAAGCCAAA	CGTCACCCCTTGTTCTTTGT	XM013276809
*g*6*pd*	GTCACCTCAACCGGGAAGTA	TGGCTGAGGACACCTCTCTT	XM013275693
*asat*	GCTTCCTTGGTGACTTGGAA	CCAGGCATCTTTCTCCAGAC	XM003451918
*alat*	CACGGTGAAGAAGGTGGAGT	GCAGTTCAGGGTAGGAGCAG	XM005476466
*gdh*	CGAGCGAGACTCCAACTACC	TGGCTGTTCTCATGATTTGC	XM003457465
Muscle metabolism			
*glut*4	GAGGATGGACATGGAGAGGA	CAGGAAAAGCGAGACTACCG	JN900493
*hk*1	CGTCGCTTAGTCCCAGACTC	TGACTGTAGCGTCCTTGTGG	XM019360229
*hk*2	CAGAGGGGAATTCGATTTGA	CCCACTCGACATTGACACAC	XM003448615
*pfkma*	AGGACCTCCAACCAACTGTG	TTTTCTCCTCCATCCACCAG	XM019349871
*pfkmb*	TTTGTGCATGAGGGTTACCA	CACCTCCAATCACACACAGG	XM003441476
*pkma*	TGACTGCTTCCTGGTCTGTG	CAGTGAAAGCTGGCAAATGA	XM005447626

a From Yang *et al*.^(40)^.

### Enzyme activity assay

The liver is the main metabolic organ, and the enzyme activity of phosphofructokinase (PFK), aspartate amino transferase (ASAT) and glutamate dehydrogenase (GDH) were detected in the liver. At weeks 36 and 41, we analysed enzymatic activities of some metabolic enzymes in the liver (two fish/replication, total = 12) including PFK, ASAT and GDH) and in the muscle (two fish/replication, total = 12) (hexokinase, HK, PFK; pyruvate kinase, PK). Tissue samples, including the liver (100 mg) or muscle (200 mg) samples, were homogenised in seven volumes of ice-cold buffer at pH 7⋅4 (50 mmol/l Tris, 5 mmol/l EDTA and 2 mmol/l DTT) and a protease inhibitor cocktail (P2714; Sigma, St Louis, MO, USA).

The homogenates of the liver samples were centrifuged at 10 000 × *g* for 20 min at 4°C, and the supernatants were used for assays of PFK (EC 2.7.1.11)^(42)^ and ASAT (EC 2.6.1.1), following the protocol of the commercial kit for enzyme assay (enzyme line, bioMérieux). In addition, the hepatic homogenates were sonicated for 1 min, followed by centrifugation at 10 000 × *g* for 20 min at 4°C for the assay of GDH (EC 1.4.1.2)^(43)^. The homogenates of the muscle samples were centrifuged at 900 × *g* for 10 min at 4°C, and the supernatants were used for analysis of HK (EC 2.7.1.1). The HK low-Km enzyme activities were assayed as previously described by Panserat *et al*.^(10)^. In addition, one more centrifugation was performed at 10 000 × *g* for 20 min at 4°C, and the supernatants were used for assays of PFK (EC 2.7.1.11)^(42)^ and PK (EC 2.7.1.40)^(11)^. Enzyme activities were measured in duplicate at 30°C (PFK) and 37°C (HK, PK, ASAT and GDH), and the nicotinamide adenine dinucleotide phosphates were determined using spectrophotometry at 340 nm. Reactions were initiated by the addition of the specific substrate, a Power Wave X (BioTek Instrument). Deionised water was used as a blank for each assay. Enzyme activity units were defined as micromoles of a substrate converted into product per minute at the assay temperature and expressed as the value per milligram of protein. Protein concentration was measured in duplicate, according to Bradford assay^(44)^, using a protein assay kit (Bio-Rad, München, Germany) with bovine serum albumin as a standard.

### Statistical analysis

Statistical analyses of the data were performed using SPSS for Windows, version 12 (SPSS Inc., Chicago, IL, USA). Independent samples *t*-test was performed to evaluate effects of the early dietary stimulus (HP/LC diet- versus LP/HC diet-fed fry) on carbohydrate and its related metabolic gene expression at weeks 1 and 4 (the end of stimulus). In addition, it was used to determine all parameters before the challenge (week 36), including growth performances, plasma metabolites, chemical composition in the liver and muscle, and the expression of genes related to carbohydrate metabolism. After the nutritional challenge (week 41), two-way factorial ANOVA was performed to determine two combination factors including the effects of early dietary stimulus and the dietary carbohydrate challenge and their interactions. When the interaction of the factors was statistically significant, one-way ANOVA following Tukey's range test was used to rank the treatment combination groups. Throughout the experiment, the effects and differences were considered significant when *P* < 0⋅05.

## Results

### Direct effects of the early HC stimulus at first feeding on growth and metabolism of adult fish

During the period of stimulus (first feeding with LP/HC or HP/LC diets for 4 weeks), there were no differences in mortality rates (*P* > 0⋅05; Table 3). Growth performances for the two groups of fish are presented in Table 3. During the stimulus period, growth response including final weight (FW), weight gain (WG), average daily gain (ADG) and specific growth rate (SGR) of fish fed the LP/HC diet was lower than that observed in fish fed the HP/LC diet (*P* < 0⋅05). Moreover, at the end of the stimulus, the body weight of fish fed the HP/LC diet at first feeding was significantly higher (1⋅4 times) than that observed in fish fed the LP/HC diet at first feeding (*P* < 0⋅05; Table 3). Note that feed intake (FI) through the end of stimulus (weeks 1 and 4) was performed at an excess amount to ensure the supply amount of diet for fry (according to commercial protocol).

**Table 3.** Growth performances of the Nile tilapia that were fed the high-protein/low-carbohydrate (HP/LC) and low-protein/high-carbohydrate (LP/HC) diets (mean ± sd, *n* 6) during the early stimulus and feeding (first 4 weeks) with a commercial diet (up to week 36)

Experimental periods	FW^3^ (g)	WG^4^ (g)	ADG^5^ (g/d)	SGR^6^ (%/d)	FI^7^ (g/d)	FCR^8^	Survival rate^9^ (%)
Week 1 (during stimulus)^1^							
HP/LC	53⋅7 ± 3⋅2 mg	44⋅1 ± 3⋅2 mg	6⋅3 ± 0⋅5 mg/d	24⋅5 ± 0⋅8	14⋅6 ± 0⋅0 mg/d	2⋅4 ± 0⋅2	No Mortality
LP/HC	52⋅3 ± 3⋅5 mg	42⋅7 ± 3⋅5 mg	6⋅1 ± 0⋅5 mg/d	24⋅2 ± 1⋅0	14⋅6 ± 0⋅0 mg/d	2⋅3 ± 0⋅2	No Mortality
*P* value^2^	0⋅514	0⋅492	0⋅490	0⋅482	1⋅000	0⋅481	–
Week 4 (end of stimulus)^1^							
HP/LC	730⋅7 ± 14⋅5 mg	721⋅1 ± 14⋅5 mg	25⋅8 ± 0⋅5 mg/d	15⋅5 ± 0⋅1	105⋅9 ± 8⋅3 mg/d	4⋅1 ± 0⋅2	No Mortality
LP/HC	517⋅1 ± 15⋅5 mg	507⋅5 ± 15⋅6 mg	18⋅1 ± 0⋅6 mg/d	14⋅2 ± 0⋅1	88⋅4 ± 6⋅6 mg/d	4⋅8 ± 0⋅3	No Mortality
*P* value^2^	<0⋅001	<0⋅001	<0⋅001	<0⋅001	0⋅002	0⋅001	–
Week 8 (4 weeks after stimulus)							
HP/LC History	3⋅40 ± 0⋅12	3⋅39 ± 0⋅11	0⋅06 ± 0⋅00	9⋅94 ± 0⋅06	0⋅15 ± 0⋅0	1⋅8 ± 0⋅1	No Mortality
LP/HC History	2⋅95 ± 0⋅11	2⋅94 ± 0⋅12	0⋅05 ± 0⋅00	9⋅70 ± 0⋅07	0⋅19 ± 0⋅0	2⋅0 ± 0⋅1	No Mortality
*P* value^2^	<0⋅001	<0⋅001	0⋅001	<0⋅001	<0⋅001	0⋅002	–
Week 24 (20 weeks after stimulus)							
HP/LC History	42⋅24 ± 0⋅63	42⋅23 ± 0⋅63	0⋅25 ± 0⋅00	4⋅99 ± 0⋅01	0⋅52 ± 0⋅01	1⋅79 ± 0⋅05	No Mortality
LP/HC History	41⋅11 ± 0⋅95	41⋅10 ± 0⋅95	0⋅24 ± 0⋅01	4⋅98 ± 0⋅01	0⋅54 ± 0⋅00	1⋅81 ± 0⋅03	No Mortality
*P* value^2^	0⋅036	0⋅032	0⋅001	0⋅036	0⋅001	0⋅393	–
Week 36 (32 weeks after stimulus)							
HP/LC History	219⋅44 ± 4⋅93	219⋅43 ± 4⋅93	0⋅87 ± 0⋅02	3⋅98 ± 0⋅00	1⋅26 ± 0⋅05	1⋅31 ± 0⋅05	97⋅7 ± 2⋅1
LP/HC History	216⋅65 ± 1⋅98	216⋅64 ± 1⋅98	0⋅86 ± 0⋅01	3⋅98 ± 0⋅01	1⋅36 ± 0⋅05	1⋅39 ± 0⋅04	98⋅2 ± 1⋅8
*P* value^2^	0⋅226	0⋅226	0⋅467	0⋅363	0⋅008	0⋅013	0⋅661

1 Note that feed intake through the end of stimulus (weeks 1 and 4) was followed according to commercial protocol. Swim-up fry were fed the experimental diet at 30 % body weight during week 1, 20 % body weight during week 2 and 10 % body weight during weeks 3–4^(18)^; therefore, over calculated FCR was presented.

2 A *t*-test analysis was used to analyse the effects of different stimulus between HP/LC and LP/HC diets.

3 Final body weight (FW).

4 Weight gain (WG) = Final body weight − Initial body weight.

5 Average daily gain (ADG) = (Final body weight − Initial body weight)/Experimental days.

6 Specific growth rate (SGR) = 100 × ((Final body weight − Initial body weight)/Experimental days).

7 Feed intake (FI) = Dry feed fed/Experimental days.

8 Feed conversion ratio (FCR) = Dry feed fed/Wet weight gain.

9 Survival rate = 100 × ((Initial number of fish − Final number of final)/Initial number of fish).

**Fig. 2.**
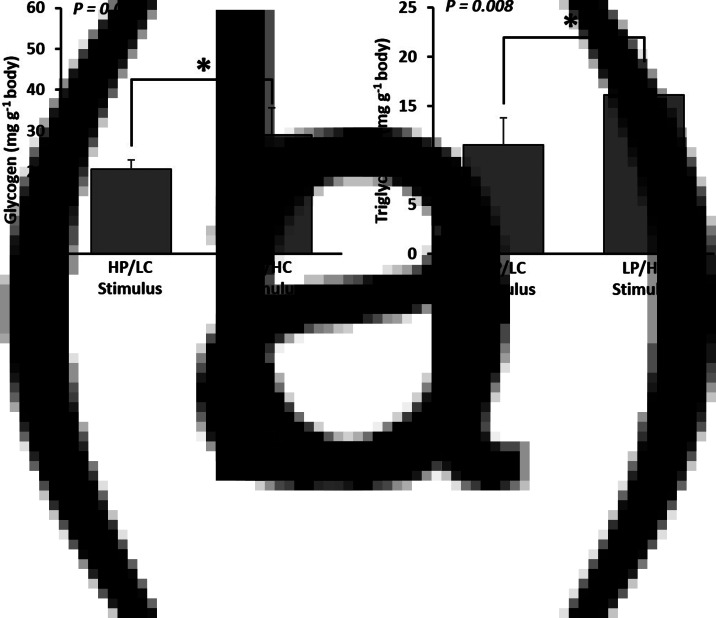
Chemical composition of fish after the early dietary stimulus. Dietary stimulus including HP/LC and LP/HC diets (history) was performed since first feeding through 4 weeks; at the end of the stimulus, whole bodies of fry (one fish/replicate; total = 6 replications) were sampled for determination of glycogen (a) and triacylglycerol (b) contents. The asterisk (*) in the bar graph indicates a significant difference (*P* < 0⋅05).

The early stimulus by intake of HC diet exerted significant effects on glucose metabolism. For instance, the direct effects of the intake of the HC diet on the expression of metabolic genes were measured at weeks 1 (during the stimulus period) and 4 (end of the stimulus period) (Table 4). After week 1 of feeding the HC diet, there were no significant differences in expression of genes involved in glycolysis in the liver, whereas the expression of several muscular glycolytic genes changed. In addition, up-regulation of *hk*2 was observed, whereas down-regulation of *pfkmb* was observed (*P* < 0⋅05). In addition, the *glut*4 mRNA level was significantly decreased in LP/HC diet-fed fry (*P* < 0⋅05). Moreover, significant down-regulation of genes involved in gluconeogenesis (*g*6*pca*1, *g*6*pca*2 and *pck*1) and amino acid catabolism (*asat* and *alat*) was also detected in LP/HC diet-fed fry (*P* < 0⋅05; Table 4). At the end of the HC stimulus (week 4), the effect of hyperglucidic stimulus was weaker than that observed after week 1 of stimulus. For example, the effects of down-regulation of genes involved in gluconeogenesis and amino acid catabolism were no more found in LP/HC diet-fed fry (*P* > 0⋅05) at the end of stimulus (Table 4). In contrast, down-regulation of *glut*4 and *pfkmb* and up-regulation of *hk*2 mRNAs were still observed in LP/HC diet-fed fry (*P* < 0⋅05). Additionally, up-regulation of *pkma* and *g*6*pd* in LP/HC diet-fed fry was detected for the first time (*P* < 0⋅05; Table 4). Finally, LP/HC diet-fed fry had higher glycogen and TAG contents than that of HP/LC diet-fed fry (Fig. 2(a) and (b)).

**Table 4.** mRNA levels of genes involved in carbohydrate metabolism in the whole body of the Nile tilapia that were fed the high-protein/low-carbohydrate (HP/LC) and low-protein/high-carbohydrate (LP/HC) diets at weeks 1 and 4 during the stimulus (mean ± sd, *n* 6)

mRNA level	Week 1 stimulus			Week 4 stimulus		
	HP/LC	LP/HC	*P* value^1^	HP/LC	LP/HC	*P* value^1^
Glycolysis						
*gck*	0⋅7 ± 0⋅4	1⋅3 ± 0⋅8	0⋅109	1⋅0 ± 0⋅4	1⋅3 ± 0⋅5	0⋅240
*pfklr*	1⋅2 ± 0⋅5	1⋅1 ± 0⋅2	0⋅660	1⋅0 ± 0⋅3	0⋅9 ± 0⋅2	0⋅422
*pklr*	1⋅6 ± 0⋅6	1⋅2 ± 0⋅2	0⋅156	1⋅2 ± 0⋅4	0⋅8 ± 0⋅1	0⋅610
Gluconeogenesis						
*g*6*pca*1	2⋅1 ± 0⋅8	0⋅3 ± 0⋅1	0⋅003	1⋅2 ± 0⋅3	1⋅0 ± 0⋅1	0⋅402
*g*6*pca*2	2⋅3 ± 0⋅8	0⋅3 ± 0⋅1	0⋅001	0⋅9 ± 0⋅3	0⋅7 ± 0⋅1	0⋅137
*pck*1	2⋅3 ± 1⋅0	0⋅3 ± 0⋅1	0⋅005	0⋅9 ± 0⋅6	1⋅0 ± 0⋅5	0⋅908
*pck*2	0⋅9 ± 0⋅6	1⋅1 ± 0⋅4	0⋅582	0⋅8 ± 0⋅2	0⋅8 ± 0⋅1	0⋅441
Lipogenesis						
*fasn*	1⋅6 ± 0⋅5	1⋅2 ± 0⋅2	0⋅175	1⋅1 ± 0⋅4	0⋅9 ± 0⋅1	0⋅191
*g*6*pd*	1⋅4 ± 0⋅5	1⋅1 ± 0⋅2	0⋅298	0⋅7 ± 0⋅1	0⋅9 ± 0⋅1	0⋅032
Amino acid catabolism						
*asat*	2⋅0 ± 0⋅7	0⋅6 ± 0⋅2	0⋅002	1⋅0 ± 0⋅3	0⋅8 ± 0⋅2	0⋅103
*alat*	1⋅6 ± 0⋅7	0⋅9 ± 0⋅2	0⋅042	1⋅0 ± 0⋅1	1⋅0 ± 0⋅1	0⋅363
*gdh*	1⋅1 ± 0⋅6	1⋅2 ± 0⋅2	0⋅628	0⋅5 ± 0⋅1	0⋅6 ± 0⋅1	0⋅287
Glucose transport and glycolysis						
*glut*4	1⋅5 ± 0⋅5	1⋅0 ± 0⋅2	0⋅040	1⋅1 ± 0⋅2	0⋅6 ± 0⋅1	0⋅002
*hk*1	1⋅4 ± 0⋅5	0⋅9 ± 0⋅2	0⋅074	1⋅1 ± 0⋅3	0⋅9 ± 0⋅1	0⋅304
*hk*2	0⋅9 ± 0⋅3	1⋅4 ± 0⋅3	0⋅004	0⋅8 ± 0⋅1	1⋅1 ± 0⋅2	0⋅016
*pfkma*	1⋅2 ± 0⋅4	1⋅1 ± 0⋅1	0⋅712	1⋅0 ± 0⋅4	0⋅9 ± 0⋅1	0⋅400
*pfkmb*	1⋅7 ± 0⋅5	0⋅9 ± 0⋅1	0⋅010	1⋅2 ± 0⋅3	0⋅8 ± 0⋅2	0⋅030
*pkma*	1⋅6 ± 0⋅7	1⋅3 ± 0⋅3	0⋅394	0⋅9 ± 0⋅1	1⋅1 ± 0⋅1	0⋅028

1 *t*-test analysis was used to analyse the effects of different stimulus between HP/LC and LP/HC diets.

Therefore, the experimental fish were maintained for evaluation of the persistent effects in adulthood. No differences were observed in mortality rates between fish previously fed LP/HC and HP/LC diets, through 36 weeks of the experimental period (*P* > 0⋅05; Table 3). During the feeding period with a commercial diet, fish with LP/HC history caught up their body weight with that of fish with HP/LC history by week 36. Moreover, FI and feed conversion ratio (FCR) of fish with LP/HC history were higher than those of fish with HP/LC history (*P* < 0⋅05; Table 3).

### Long-term effects of early HC stimulus in relation to the high dietary carbohydrate challenge in adult fish

**Fig. 3.**
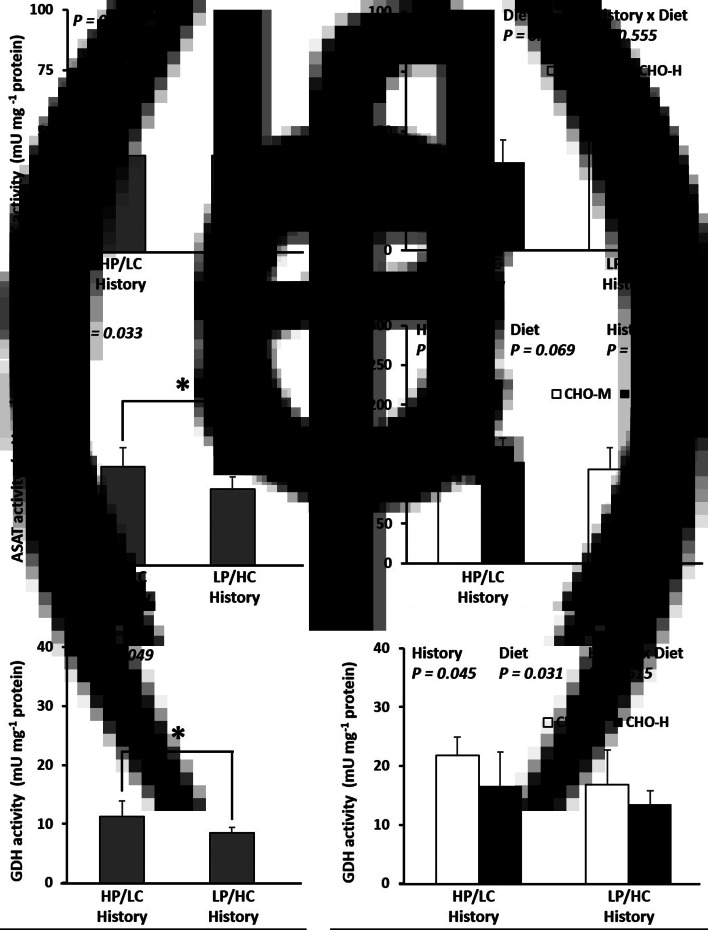
Enzyme activities (mU/mg protein) in the livers of the Nile tilapia that were early fed the HP/LC diet or the LP/HC diet (history) and challenged in adulthood with CHO-M or CHO-H diets. PFK (a,b), which is involved in glycolysis, and ASAT (c,d) and GDH (e,f) in amino acid catabolism were analysed in the liver samples. At weeks 36–41, the fish were subjected to a challenge test with different dietary carbohydrate levels (37 % carbohydrates, CHO-M; 67 % carbohydrates, CHO-H). Before the challenge test, the fish were sampled to determine the effect of stimulus diet history on the PFK, ASAT and GDH activities in the liver (a,c,e). The asterisk (*) in the bar graph indicates a significant difference (*P* < 0⋅05). After the challenge test (week 41), the combination effects of stimulus history and challenge diet on the PFK, ASAT and GDH activities in the liver were examined (b,d,f). Data are presented as the mean ± standard deviation (sd) (*n* 6). Two-way ANOVA was used to analyse the effects of stimulus diets (history), challenge diets (diet) and their interaction (history × diet).

**Fig. 4.**
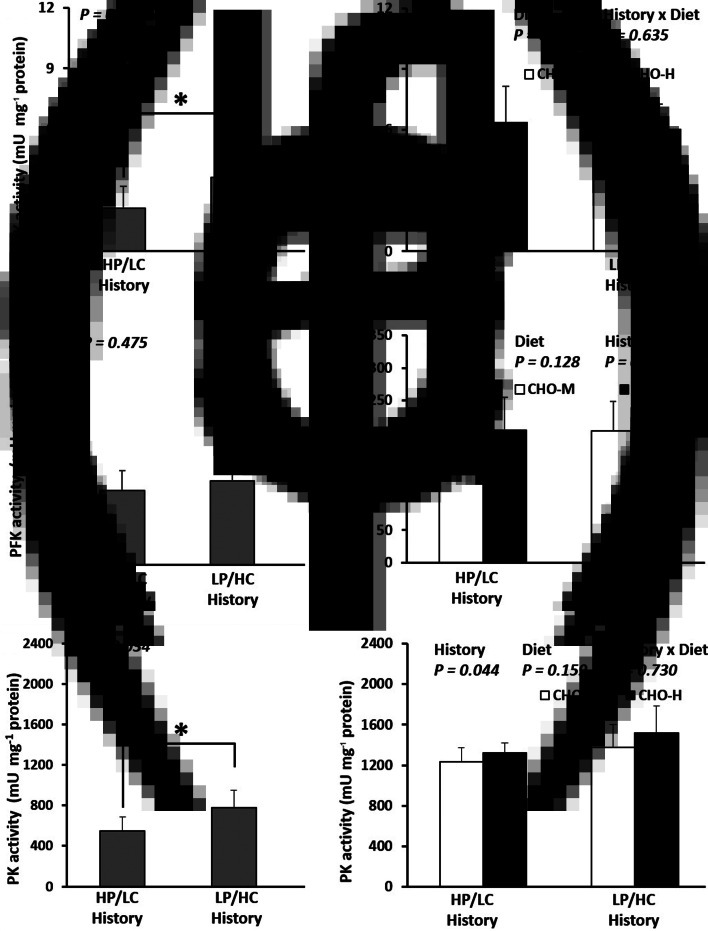
Enzyme activities (mU/mg protein) in the muscles of the Nile tilapia that were early fed the HP/LC diet or the LP/HC diet (history) and challenged in adulthood with CHO-M or CHO-H diets. HK (a,b), PFK (c,d) and PK (e,f), which are involved in glycolysis, were analysed in the muscle samples. At weeks 36–41, the fish were subjected to a challenge test with different dietary carbohydrate levels (37 % carbohydrates, CHO-M; 67 % carbohydrates, CHO-H). Before the challenge test, the fish were sampled to determine the effect of stimulus diet history on the HK, PFK and PK activities in the muscle (a,c,e). The asterisk (*) in the bar graph indicates a significant difference (*P* < 0⋅05). After the challenge test (week 41), the combination effects of stimulus history and challenge diet on the HK, PFK and PK activities in the muscle were examined (b,d,f). Data are presented as the mean ± standard deviation (sd) (*n* 6). Two-way ANOVA was used to analyse the effects of stimulus diets (history), challenge diets (diet) and their interaction (history × diet).

To examine the existence of nutritional programming in relation to the early HC stimuli history, fish with both HP/LC history and LP/HC history were challenged with a CHO-H diet at weeks 37–41. Results of the two effects (history and challenge) and their interaction on growth performance (Table 5), hepatic and muscular composition (Table 6), plasma metabolites (Table 7) and mRNA levels of genes involved in intermediary metabolism (Table 8; Figs. 3 and 4) were obtained.

**Table 5.** Growth performance of the Nile tilapia fed with high-protein/low-carbohydrate (HP/LC) and low-protein/high-carbohydrate (LP/HC) at before (36 wps) and after (41 wps) challenging them with the medium-carbohydrate diet (CHO-M) and the high-carbohydrate diet (CHO-H) for 4 weeks (mean ± sd, *n* 6)

Parameters	HP/LC history		LP/HC history		*P* value^1^		
	CHO-M	CHO-H	CHO-M	CHO-H	History	Diet	Interactions
IW^2^ (g)	211⋅1 ± 2⋅2	211⋅7 ± 2⋅0	210⋅1 ± 0⋅9	211⋅3 ± 2⋅1	0⋅338	0⋅257	0⋅700
FW^3^ (g)	322⋅3 ± 7⋅5	300⋅9 ± 6⋅5	331⋅2 ± 7⋅6	316⋅5 ± 8⋅1	<0⋅001	<0⋅001	0⋅293
WG^4^ (g)	111⋅2 ± 7⋅4	89⋅2 ± 7⋅6	121⋅2 ± 6⋅9	105⋅3 ± 9⋅8	<0⋅001	<0⋅001	0⋅371
ADG^5^ (g/d)	3⋅4 ± 0⋅2	2⋅7 ± 0⋅2	3⋅7 ± 0⋅2	3⋅2 ± 0⋅3	<0⋅001	<0⋅001	0⋅378
SGR^6^ (%/d)	1⋅3 ± 0⋅1	1⋅1 ± 0⋅1	1⋅4 ± 0⋅1	1⋅2 ± 0⋅1	<0⋅001	<0⋅001	0⋅372
FI^7^ (g/d)	3⋅5 ± 0⋅2	3⋅4 ± 0⋅4	3⋅5 ± 0⋅3	3⋅3 ± 0⋅3	0⋅958	0⋅359	0⋅577
FCR^8^	1⋅0 ± 0⋅1	1⋅3 ± 0⋅2	1⋅0 ± 0⋅1	1⋅1 ± 0⋅2	0⋅034	0⋅013	0⋅214
Survival rate^9^ (%)	91⋅7 ± 9⋅1	94⋅4 ± 8⋅6	94⋅4 ± 8⋅6	94⋅4 ± 8⋅6	0⋅701	0⋅701	0⋅701

1 Two-way ANOVA was used to analyse the effects of dietary stimulus (history), challenging diet (diet) and their interaction (history × diet).

2 Initial body weight (IW).

3 Final body weight (FW).

4 Weight gain (WG) = Final body weight − Initial body weight.

5 Average daily gain (ADG) = (Final body weight − Initial body weight)/Experimental days.

6 Specific growth rate (SGR) = 100 × ((Final body weight − Initial body weight)/Experimental days).

7 Feed intake (FI) = Dry feed fed/Experimental days.

8 Feed conversion ratio (FCR) = Dry feed fed/Wet weight gain.

9 Survival rate = 100 × ((Initial number of fish − Final number of final)/Initial number of fish).

**Table 6.** Proximate composition of the Nile tilapia fed the high-protein/low-carbohydrate (HP/LC) and low-protein/high-carbohydrate (LP/HC) diets before (36 wps) and after (41 wps) challenging them with the medium-carbohydrate diet (CHO-M) and the high-carbohydrate diet (CHO-H) for 4 weeks (mean ± sd, *n* 6)

Parameter tissue	History (before challenge)			HP/LC history		LP/HC history		*P* value^1^		
	HP/LC	LP/HC	*P* value	CHO-M	CHO-H	CHO-M	CHO-H	History	Diet	Interactions
LIVER (g/kg)										
Protein	102⋅4 ± 2⋅2	109⋅6 ± 2⋅1	0⋅001	103⋅0 ± 3⋅2^a^	94⋅2 ± 2⋅3^c^	97⋅2 ± 3⋅3^bc^	98⋅9 ± 2⋅6^ab^	0⋅674	0⋅007	<0⋅001
Fat	42⋅7 ± 0⋅4	43⋅6 ± 0⋅6	0⋅007	30⋅0 ± 2⋅3^d^	46⋅1 ± 3⋅2^b^	36⋅7 ± 2⋅5^c^	62⋅8 ± 2⋅0^a^	<0⋅001	<0⋅001	<0⋅001
Ash	13⋅9 ± 0⋅1	14⋅0 ± 0⋅3	0⋅466	14⋅7 ± 0⋅5	14⋅2 ± 1⋅1	14⋅1 ± 1⋅0	14⋅7 ± 0⋅7	0⋅962	0⋅955	0⋅150
HSI^2^ (%)	4⋅0 ± 0⋅1	4⋅0 ± 0⋅3	0⋅661	3⋅5 ± 0⋅1	4⋅0 ± 0⋅4	3⋅6 ± 0⋅3	4⋅4 ± 0⋅3	0⋅073	<0⋅001	0⋅168
Glycogen (mg/g)	209⋅7 ± 4⋅4	236⋅8 ± 19⋅2	0⋅007	210⋅8 ± 41⋅8	287⋅0 ± 45⋅8	327 ⋅0 ± 31⋅1	342⋅3 ± 29⋅3	0⋅007	<0⋅001	0⋅061
Muscle (g/kg)										
Protein	174⋅4 ± 1⋅8	171⋅8 ± 2⋅8	0⋅086	173⋅6 ± 4⋅7^ab^	167⋅2 ± 4⋅5^b^	178⋅7 ± 4⋅9^a^	159⋅9 ± 1⋅9^c^	0⋅530	<0⋅001	0⋅002
Fat	17⋅1 ± 1⋅5	15⋅5 ± 1⋅0	0⋅063	23⋅3 ± 3⋅5	41⋅3 ± 2⋅4	25⋅3 ± 3⋅7	43⋅5 ± 1⋅3	0⋅089	<0⋅001	0⋅899
Ash	14⋅1 ± 0⋅2	14⋅3 ± 0⋅2	0⋅117	15⋅4 ± 0⋅4	15⋅8 ± 0⋅9	15⋅9 ± 0⋅6	16⋅0 ± 0⋅5	0⋅165	0⋅310	0⋅671
Glycogen (mg/g)	6⋅4 ± 0⋅3	7⋅5 ± 0⋅8	0⋅008	6⋅8 ± 2⋅0	8⋅2 ± 0⋅9	8⋅4 ± 1⋅2	9⋅2 ± 0⋅8	0⋅023	0⋅059	0⋅552

1 Two-way ANOVA was used to analyse the effects of dietary stimulus (history), challenge diet (diet) and their interaction (history × diet). One-way ANOVA following Tukey's range test was used to rank the treatment combination groups when the interaction of the factors was statistically significant. Different letters indicate significant differences in the mean values for four combination groups (*P* < 0⋅05).

2 Hepatosomatic index (HSI) = 100 × (Liver weight/Body weight).

**Table 7.** Plasma metabolites of the Nile tilapia fed with high-protein/low-carbohydrate (HP/LC) and low-protein/high-carbohydrate (LP/HC) diets before (36 wps) and after (41 wps) challenging them with the medium-carbohydrate diet (CHO-M) and the high-carbohydrate diet (CHO-H) for 4 weeks (mean ± sd, *n* 6)

Parameters	History (before challenge)			HP/LC history		LP/HC history		*P* value^1^		
	HP/LC	LP/HC	*P* value	CHO-M	CHO-H	CHO-M	CHO-H	History	Diet	Interactions
Glucose (mM)	3⋅6 ± 0⋅6	4⋅2 ± 0⋅9	0⋅185	4⋅3 ± 0⋅3	4⋅2 ± 0⋅5	4⋅9 ± 0⋅4	5⋅2 ± 0⋅3	<0⋅001	0⋅515	0⋅127
Triacylglycerol (mM)	3⋅0 ± 0⋅5	2⋅8 ± 0⋅5	0⋅549	1⋅6 ± 0⋅3	2⋅0 ± 0⋅3	1⋅6 ± 0⋅5	2⋅4 ± 0⋅5	0⋅247	0⋅002	0⋅275
BUN (mM)	1⋅1 ± 0⋅1	1⋅1 ± 0⋅1	0⋅616	1⋅2 ± 0⋅1	0⋅9 ± 0⋅1	1⋅1 ± 0⋅1	0⋅8 ± 0⋅1	0⋅007	<0⋅001	0⋅294

BUN, blood urea nitrogen.

1 Two-way ANOVA was used to analyse the effects of dietary stimulus (history), challenging diet (diet) and their interaction (history × diet).

**Table 8.** mRNA levels of genes involved in carbohydrate metabolism in the liver and muscles of fish fed with high-protein/low-carbohydrate (HP/LC) and low-protein/high-carbohydrate (LP/HC) diets before (36 wps) and after (41 wps) challenging them with the medium-carbohydrate diet (CHO-M) and the high-carbohydrate diet (CHO-H) for 4 weeks (mean ± sd, *n* 6)

Parameter tissue	History (before challenge)			HP/LC history		LP/HC history		*P* value^1^		
	HP/LC	LP/HC	*P* value	CHO-M	CHO-H	CHO-M	CHO-H	History	Diet	Interactions
Liver glycolysis										
*gck*	0⋅5 ± 0⋅2	1⋅5 ± 0⋅8	0⋅027	0⋅9 ± 0⋅6	0⋅8 ± 0⋅3	0⋅7 ± 0⋅4	0⋅6 ± 0⋅2	0⋅259	0⋅569	0⋅947
*pfklr*	1⋅2 ± 0⋅6	1⋅2 ± 0⋅9	0⋅888	0⋅7 ± 0⋅2^a^	0⋅4 ± 0⋅2^b^	0⋅3 ± 0⋅1^b^	0⋅3 ± 0⋅1^b^	0⋅009	0⋅057	0⋅046
*pklr*	1⋅0 ± 0⋅3	1⋅5 ± 0⋅7	0⋅088	1⋅0 ± 0⋅6	0⋅8 ± 0⋅5	1⋅0 ± 0⋅6	0⋅6 ± 0⋅2	0⋅418	0⋅122	0⋅503
Liver gluconeogenesis										
*g*6*pca*1	1⋅4 ± 0⋅7	0⋅9 ± 0⋅6	0⋅226	0⋅8 ± 0⋅5	0⋅6 ± 0⋅4	0⋅6 ± 0⋅4	0⋅4 ± 0⋅2	0⋅176	0⋅176	0⋅937
*g*6*pca*2	1⋅3 ± 1⋅0	0⋅6 ± 0⋅5	0⋅164	0⋅5 ± 0⋅3	0⋅4 ± 0⋅4	0⋅5 ± 0⋅3	0⋅2 ± 0⋅1	0⋅297	0⋅100	0⋅332
*pck*1	0⋅7 ± 0⋅4	0⋅6 ± 0⋅3	0⋅617	0⋅08 ± 0⋅07	0⋅78 ± 0⋅90	0⋅03 ± 0⋅06	0⋅07 ± 0⋅14	0⋅055	0⋅064	0⋅093
*pck*2	0⋅9 ± 0⋅5	0⋅4 ± 0⋅4	0⋅078	1⋅1 ± 0⋅7	0⋅5 ± 0⋅4	0⋅9 ± 0⋅6	0⋅6 ± 0⋅4	0⋅794	0⋅054	0⋅584
Liver lipogenesis										
*fasn*	0⋅7 ± 0⋅4	1⋅2 ± 0⋅6	0⋅158	0⋅5 ± 0⋅3	0⋅9 ± 0⋅8	0⋅4 ± 0⋅2	0⋅9 ± 0⋅3	0⋅979	0⋅018	0⋅636
*g*6*pd*	1⋅0 ± 0⋅3	1⋅6 ± 0⋅6	0⋅056	0⋅5 ± 0⋅3	0⋅8 ± 0⋅3	0⋅3 ± 0⋅2	1⋅0 ± 0⋅4	0⋅768	0⋅001	0⋅180
Liver amino acid catabolism										
*asat*	1⋅3 ± 0⋅5	0⋅5 ± 0⋅3	0⋅049	0⋅6 ± 0⋅3	0⋅8 ± 0⋅4	0⋅5 ± 0⋅2	0⋅4 ± 0⋅3	0⋅046	0⋅727	0⋅311
*alat*	1⋅3 ± 0⋅4	0⋅8 ± 0⋅4	0⋅048	1⋅4 ± 0⋅4	0⋅7 ± 0⋅4	0⋅7 ± 0⋅3	0⋅5 ± 0⋅3	0⋅005	0⋅007	0⋅137
*gdh*	0⋅9 ± 0⋅3	1⋅4 ± 0⋅5	0⋅053	1⋅1 ± 0⋅3	0⋅8 ± 0⋅5	0⋅8 ± 0⋅3	0⋅6 ± 0⋅3	0⋅158	0⋅096	0⋅684
Glucose transport and Muscle metabolism										
*glut*4	0⋅7 ± 0⋅2	1⋅1 ± 0⋅5	0⋅152	0⋅8 ± 0⋅2^ab^	0⋅6 ± 0⋅3^b^	0⋅7 ± 0⋅4^ab^	1⋅4 ± 0⋅8^a^	0⋅060	0⋅157	0⋅030
*hk*1	0⋅9 ± 0⋅2	1⋅5 ± 0⋅4	0⋅009	0⋅9 ± 0⋅3	1⋅0 ± 0⋅5	0⋅8 ± 0⋅3	1⋅4 ± 0⋅7	0⋅605	0⋅092	0⋅207
*hk*2	1⋅0 ± 0⋅4	1⋅2 ± 0⋅2	0⋅250	1⋅2 ± 0⋅4	0⋅9 ± 0⋅2	0⋅6 ± 0⋅5	1⋅2 ± 0⋅6	0⋅411	0⋅388	0⋅040
*pfkma*	0⋅7 ± 0⋅3	0⋅9 ± 0⋅4	0⋅369	1⋅0 ± 0⋅2	0⋅6 ± 0⋅2	0⋅6 ± 0⋅4	1⋅0 ± 0⋅5	0⋅865	0⋅873	0⋅019
*pfkmb*	1⋅2 ± 0⋅4	0⋅8 ± 0⋅1	0⋅044	1⋅2 ± 0⋅4	1⋅2 ± 0⋅5	0⋅5 ± 0⋅1	0⋅5 ± 0⋅2	<0⋅001	0⋅909	0⋅957
*pkma*	0⋅5 ± 0⋅2	0⋅7 ± 0⋅2	0⋅030	1⋅2 ± 0⋅3	1⋅0 ± 0⋅5	0⋅7 ± 0⋅2	1⋅1 ± 0⋅3	0⋅179	0⋅357	0⋅035

1 Two-way ANOVA was used to analyse the effects of dietary stimulus (history), challenging diet (diet) and their interaction (history × diet). One-way ANOVA following Tukey's range test was used to rank the treatment combination groups when the interaction of the factors was statistically significant. Different letters indicate significant differences in the mean values for four combination groups (*P* < 0⋅05).

Regarding growth performance, the results indicated that independently of the two diets for the challenge, higher final body weight, ADG, and SGR and lower FCR were observed in fish with LP/HC history than in fish with HP/LC history (*P* < 0⋅05; Table 5). As expected, irrespective of the dietary stimulus, the CHO-H diet led to lower growth performance compared with that in fish fed the CHO-M diet (*P* < 0⋅05; Table 5).

The effects of the early HC stimulus and the high dietary carbohydrate final challenge on chemical composition in the liver and muscle are presented in Table 6. Before the dietary challenge, early high-carbohydrate (LP/HC) history is associated with permanent effects on increased protein, lipid and glycogen in the liver (*P* < 0⋅05). Moreover, for muscle composition (Table 6), early HC feeding history led to significantly increased glycogen in adult tilapia (*P* < 0⋅05). Subsequently, after the dietary challenge, early HC history was associated with an increase in hepatic fat and glycogen in both the liver and muscle (*P* < 0⋅05). Moreover, dietary CHO-H led to significantly decreased hepatic protein contents (*P* < 0⋅05) and increased fat and glycogen levels with higher HSI (*P* < 0⋅05). Finally, fish fed CHO-H diet had lower levels of protein and higher levels of fat in the muscle (*P* < 0⋅05). Interaction effects between nutritional history (stimulus) and the final challenge were also found for the hepatic fat and protein in both the liver and muscle. Indeed, LP/HC diet-fed fish fed the CHO-H diet had higher fat in the liver and lower protein content in the muscle (*P* < 0⋅05; Table 6).

However, just before the final dietary challenge, early HC history had no significant effects on the plasma metabolites (glucose, triacylglycerol and BUN) (*P* > 0⋅05; Table 7). In contrast, fish with LP/HC history had higher plasma glucose and lower BUN after the final dietary challenge irrespective of the diets (*P* < 0⋅05). Finally, fish fed the CHO-H diet exhibited an increase in plasma TAG and a decrease in BUN (*P* < 0⋅05; Table 7).

Before the dietary challenge, fish with an early HC history had higher mRNA levels of hepatic *gck* and muscle *hk*1 and *pkma* and higher enzymatic activities for the latter two (*P* < 0⋅05; Table 8; Fig. 4(a) and (e)). In addition, there was down-regulation of hepatic *asat* (mRNA and ASAT activity), *alat* (mRNA) and *gdh* (not mRNA but glutamate dehydrogenase activity alone) as well as muscle *pfkmb* (only mRNA but not phosphofructokinase activity) (*P* < 0⋅05; Table 8; Figs. 3 and 4). The transcripts of other genes such as *pfklr*, *pklr*, *g*6*pca*1, *g*6*pca*2, *pck*1, *pck*2, *fasn*, *g*6*pd*, *glut*4, *hk*2, *pfkma* and hepatic PFK enzyme activity remained unchanged (*P* > 0⋅05). When fish with HP/LC history and LP/HC history had been subjected to a final dietary challenge with CHO-M and CHO-H diets, the effects of early HC feeding were always detectable as reflected by the down-regulation of *asat* (mRNA and ASAT activity), *alat* (mRNA) and *pfkmb* (mRNA but not PFK activity) (*P* < 0⋅05; Table 8; Figs. 3 and 4). However, only fish with early HC history was associated with a decrease in hepatic *gdh* and an increase in muscular PK activities (*P* < 0⋅05; Table 8; Figs. 3 and 4). Additionally, it must be noted that hepatic *pfklr* mRNA decreased, whereas PFK activity increased (*P* < 0⋅05; Table 8; Fig. 3(b)). There were no significant differences in expressions of *gck*, *pklr*, *g*6*pca*1, *g*6*pca*2, *pck*1, *pck*2, *fasn*, *g*6*pd*, *glut*4, *hk*1 (mRNA and its activity), *hk*2 (mRNA and its activity) and *pfkma* (mRNA and its activity) (*P* > 0⋅05; Table 8; Fig. 4). For the specific effects of the dietary challenge, fish fed the CHO-H diet indicated increased expression of *fasn* and *g*6*pd* and decreased mRNA level of *alat* (*P* < 0⋅05; Table 8). In addition, CHO-H diet resulted in reduction of PFK and GDH activities (*P* < 0⋅05; Fig. 3(b) and (f)). The effects of the dietary challenge were not detectable for other gene expressions at mRNA levels (*gck*, *pfklr*, *pklr*, *g*6*pca*1, *g*6*pca*2, *pck*1, *pck*2, *asat*, *gdh*, *glut*4, *hk*1, *hk*2, *pfkma*, *pfkmb* and *pkma*) and enzyme activities (hepatic ASAT and muscle HK, PFK and PK) (*P* > 0⋅05; Table 8; Fig. 3). Interactions between history and dietary challenge were observed for mRNA levels of *pfklr*, *glut*4, *hk*2, *pfkma* and *pkma*. In fact, HP/LC diet-fed fish fed on CHO-M diet had the highest *pfklr* mRNA level. Meanwhile, the highest *glut*4 mRNA level was observed in LP/HC diet-fed fish fed CHO-H diet (*P* < 0⋅05; Table 8).

## Discussion

The concept of nutritional programming has been recently applied for better use of new feeds in aquaculture^(2,9)^. Because early dietary feeding is a potential window of metabolic plasticity and, therefore, has become the most popular early nutritional intervention, an HC diet during first feeding was provided to Nile tilapia to obtain original data. Moreover, several reports have described nutritional programming through early fry feeding in different fish species, mainly carnivorous species^(19,20,21,22,24–26)^. Therefore, our study provides original data on the long-term effect of feeding high levels of carbohydrates at first feeding on the metabolism and growth of adult Nile tilapia, an omnivorous fish species.

### Direct effects of the early HC stimulus at first feeding in fish on growth and metabolism

As expected, HP/LC diet-fed fry had higher WG (1⋅4-fold) than LP/HC diet-fed fry. It must be noted that there were no detrimental effects (decrease of survival and malformations) on the vital development of the fish.

The nutritional programming concept generally hypothesizes that animals receive stimulus in early life, which are subsequently recorded and remembered, and its effects are consequently revealed in later life^(3,4)^. Therefore, before analysing the nutritional programming concept, we needed to ensure that the Nile tilapia well received the early dietary stimulus. At the end of the stimulus period, our results indicated higher whole body glycogen and TAG contents in LP/HC diet-fed fish, which suggest much higher intake of carbohydrates in this experimental fish. Furthermore, molecular data on intermediary metabolism confirm this observation at the beginning (week 1) and at the end (week 4) of the stimulus. Overall, the HC diet stimulus suppressed muscular glucose transporter *glut*4 and inhibited several enzymes involved in gluconeogenesis (*g*6*pca*1, *g*6*pca*2 and *pck*1) and amino acid catabolism (*asat* and *alat*). Although the modulatory effect was not detectable for hepatic glycolysis, it was detected for muscular glycolysis (*hk*2 and *pfkmb*). Note that HC stimulus at week 4 induced lipogenetic *g*6*pd*. These findings suggest that fish responded to early stimuli, i.e. hyperglucidic diet is associated with an inhibition of gluconeogenesis, amino acid catabolism, and glucose transporter and induction of lipogenesis, as it has been previously observed in other fish species, such as European seabass, sturgeon, zebrafish and gilthead seabream, with carbohydrates at first feeding as well as in Nile tilapia acutely treated with glucose injection into yolk reserves^(19,20,21,23,25,26,29,45)^. Notably, although not well understood, our results indicate that at the molecular level, dietary LP/HC from the first feeding influenced several carbohydrate metabolism-related pathways and the effects of stimulus at week 1 seemed to be stronger than those at week 4. Combining all findings together, a hyperglucidic stimulus at early feeding with the HC diet are effective in changing intermediary metabolism (particularly glucose metabolism). However, the question was whether after the stimulus, this early dietary stimulus could be remembered by the Nile tilapia later in life.

### Positive long-term effects of the early HC stimulus on growth and metabolism in adult fish

After the stimulus, LP/HC diet-fed fish exhibited higher growth performance through an increase of FI, which enabled their body weight to catch up with that of HP/LC diet-fed fish at week 36 (just before the dietary challenge), demonstrating the existence of compensatory mechanisms that have been previously observed in tilapia^(46)^. Our results indicated that the early HC diet history was associated with persistent effects on hepatic biochemical composition, including higher levels of protein, lipid and glycogen contents and muscle glycogen in adult tilapia. Moreover, glucose metabolic pathway was also modified at the molecular level. In fish fed the early HC diet, there was up-regulation of glycolytic *gck*, *hk* and *pkma* and down-regulation of amino acid catabolism (*asat* and *alat*). All these data were similar to those observed by us in a previous study on juvenile tilapia, which were injected with glucose in the yolk^(29)^. Taken together, the effects of early hyperglucidic intervention (through either glucose injection into the yolk or early feeding with carbohydrates) seem to have strong impacts later in the life of the tilapia, particularly by inducing glycolysis and lipogenesis and suppressing amino acid catabolism. In other words, the tilapia seems to have better capacity to use glucose by sparing proteins (from amino acid catabolism).

### Positive long-term effects of the early HC stimulus irrespective of the final dietary challenge on growth and metabolism of adult fish

Different dietary carbohydrate and protein levels inevitably resulted in different micronutrients. In the present study, all experimental diets (both stimulus and challenge period) were supplemented with a fish premix to ensure all essential microelements meet the requirement of vital functions and normal growth. Although there might be the effects of micronutrients from contents of experimental diets and/or interaction of carbohydrate and microelements, it was probably not significantly associated with carbohydrate metabolism at the molecular level and growth response. During weeks 37–41, all fish were subjected to a dietary challenge with either CHO-H or CHO-M diet. Consistent with our previous data, the growth performance was better with CHO-M diet than with CHO-H diet^(17,18)^. Notably, the early HC diet stimulus, irrespective of the challenge diet, has a positive effect on the growth performance of adult tilapia. These findings are similar to the previous findings on the effects of early glucose injection on the improvement of growth performance of juvenile Nile tilapia^(29)^. In contrast, no improvement of growth performance was observed with early hyperglucidic stimulus in other fish species such as zebrafish, gilthead seabream, rainbow trout and European sea bass^(19,20,23,24,25,26,33)^. This effect of early feeding could be dependent on fish species.

Regarding the effect of the CHO-H diet, we observed a reduction of hepatic protein and plasmatic BUN, an elevation of hepatic fat and plasma triacylglycerol, and an increase in HSI. These findings were consistent with those reported in previous studies^(18,47,48)^. Notably, intake of an HC diet did not significantly increase postprandial glycaemia, suggesting a high ability of Nile tilapia to regulate glucose homeostasis when fed with carbohydrates, as observed previously in omnivorous fish^(13,16,17,18)^. Indeed, HC intake is associated with higher lipogenic gene expressions (*fasn* and *g*6*pd*), lower gene expressions for amino acid catabolism (*alat*), lower enzyme activities (GDH) for amino acid catabolism and higher enzyme activities for glycolysis (PFK) in the liver. All these expected regulations by dietary carbohydrates can explain the better use of dietary carbohydrates in tilapia (an omnivorous fish species) than in rainbow trout (a carnivorous fish species) with poor regulation of lipogenesis and amino acid catabolism in the liver^(13,16,17,18)^.

Regarding the effect of the early LP/HC diet stimulus (the programming effect), some interesting metabolic data have been observed that could be related to better growth performance. First, lower levels of hepatic and muscle protein and higher levels of lipid and glycogen in the liver and the muscle as well as lower plasma BUN and higher glycaemia strongly suggest that the glucose, lipid and amino acid metabolism modifications in adult tilapia are linked to the early nutritional stimulus (i.e. the LP/HC stimulus). These findings about plasma and metabolite compositions are similar to those in previous reports on tilapia programmed through glucose injection in the yolk^(29)^ but not to those on other fish species, such as gilthead seabream, rainbow trout and sturgeon, for which no significance was found for these parameters^(19,20,25,33)^. Second, it was interesting to analyse the metabolism at the enzymatic and molecular levels in relation to the nutritional history. Moreover, early experience of tilapia with HC diet is associated with lower levels of amino acid catabolism (down-regulation of *asat*; both mRNA and enzyme, *alat*; mRNA, *gdh*; enzyme) and higher levels of glycolysis (hepatic PFK and muscle PK activities even though their mRNA levels did not follow the same trends). These findings were in line with the reports described previously in juvenile tilapia stimulated with direct glucose injection^(29)^ and in omnivorous zebrafish, which were fed early with high levels of carbohydrates^(26)^. However, this is different to what was observed in carnivorous fish; for example, in rainbow trout fed at first feeding with high levels of carbohydrates, no permanent effects on hepatic glucose metabolism were observed in juvenile and only muscle glycolysis and transport were changed^(20,33)^. In contrast, in gilthead seabream, there were no obvious effects on glycolysis, gluconeogenesis and lipogenesis^(24,25)^. Taken together, our study proves without ambiguity that early feeding can have strong and long-term impacts on nutrient use and growth performance of the Nile tilapia. Further investigation should be conducted to explore whether epigenetics (at the levels of specific candidate genes and/or at the global epigenome) are involved in the regulation of nutritional programming. The nutritional programming concept of dietary carbohydrate for modulation of fish metabolism could impact the shift of fish nutrition strategy to modulate particular metabolism for the efficient use of a plant-based diet, which involves an environmentally friendly diet. Nevertheless, its effects on flesh quality for human consumption remain to be investigated.

## Conclusion

In conclusion, early nutrition can be involved in permanent changes in metabolism later in the tilapia's life, as it is well described in mammals^(3,4)^. Further studies are required to better understand the mechanisms (such as epigenetics) at the origin of these observations.
